# The pillars of the sea: strategies to achieve successful marine citizen science programs in the Mediterranean area

**DOI:** 10.1186/s12862-024-02289-0

**Published:** 2024-07-19

**Authors:** Martina Coppari, Camilla Roveta, Cristina Di Camillo, Joaquim Garrabou, Serena Lucrezi, Torcuato Pulido Mantas, Carlo Cerrano

**Affiliations:** 1 Department of Life and Environmental Sciences (DiSVA), Università Politecnica delle Marche, Via Brecce Bianche s.n.c, Ancona, 60131 Italy; 2 National Biodiversity Future Center (NBFC), Palermo, Italy; 3 Institut de Ciències del Mar (CSIC), Pg. Marítim de la Barceloneta, 37, Ciutat Vella, Barcelona, 08003 Spain; 4 Tourism Research in Economics, Environs and Society (TREES), North-West University, 11 Hoffman Street, Potchefstroom, 2531 South Africa; 5 Stazione Zoologica Anton Dohrn, Villa Comunale, Via Francesco Caracciolo s.n.c, Napoli, 80122 Italy; 6 Fano Marine Center, Viale Adriatico 1, Pesaro-Urbino, 61032 Fano Italy

**Keywords:** Public engagement, Conservation, Stakeholders, Marine environments, Community-based monitoring

## Abstract

Marine ecosystems are facing a dramatic loss of biodiversity worldwide, together with a widespread collapse of habitats and their functionality. In this context, Marine Citizen Science (MCS) can be a powerful tool to monitor these changes over time. The flowering of very well-structured international projects is strengthening the scientific credibility of MCS data, especially when data are collected after specifically designed training programs and shared in public user-friendly repositories. Here we present a new perspective on the use of MCS in the Mediterranean area, along with the main benefits for the stakeholders (i.e., diving centers, trainers, and policymakers) and the users (i.e., divers), resumed in three pillars: Pillar I – MCS as a tool for the site valorization; Pillar II – MCS as a new career opportunity for graduated students; Pillar III – MCS as a business opportunity for diving centers. In the frame of the Quintuple Helix Approach, for which there is a strong need of a socioecological transition of the society and economy, we show how MCS can be a win-win-win solution for all the actors involved, providing the vision for new and highly qualified job and business opportunities for the diving sector.

## Background

Citizen Science (CS) (i.e., the involvement of citizens in the collection of scientific data) has a long history [1], with the first collaborations between volunteers and scientists mainly related with terrestrial and freshwater topics (e.g., birdwatching, freshwater biodiversity, with a particular focus on introduced fish species, and environmental quality monitoring) [2–6].

With the advent of the web and social networks, CS started to play its full potential [7–9]. Many benefits in its use have been highlighted, not only for governance and policy but also from a scientific perspective: the direct involvement of citizens expands the spatial and temporal ranges of data collection and increases the society’s awareness and knowledge of the environment and the threats mining its stability [10, 11].

In the last 50 years, recreational SCUBA diving has become one of the most attractive leisure activities in the tourist sector [12]. Following the exponential growth of SCUBA diving, Marine Citizen Science (MCS) became a hot topic worldwide, although still not comparable to its terrestrial counterparts [8, 13, 14], due to the inaccessibility of some marine environments, the need for special diving skills to reach them, and a higher number of steps to share the collected data [10, 15]. In the past, most of the MCS projects were mainly focused on tropical areas as fruitful tourist attractions, representing an important source of income for diving centers [16]. More recently, a growing interest in countries facing temperate seas was recorded, making possible the large-scale monitoring of some of their most charismatic habitats (e.g., kelp forests, seagrass meadows, coralligenous assemblages) [17].

In the Mediterranean Sea, since the late ‘90s, several European projects have risen at a basin level (e.g., Green Bubbles, CIGESMED, Interreg Med MPA Engage), with the implementation of various MCS initiatives (e.g., Reef Check Italia, Observadores del Mar, Reef Alert Network, DORIS, iNaturalist) for the monitoring of bioherms, benthic organisms and fish fauna, either by visual assessments or photographic contests [11, 18, 19]. Four important aspects characterize effective and successful MCS initiatives: (i) the development of web platforms allowing for the immediate uploading and sharing of collected data, making them available to the general public and the scientific community; (ii) the intensive use of social media to easily exchange information and opinions (e.g., species identification), and to promote initiatives or events for the engagement of a larger number of volunteers [20–23]; (iii) the implementation of training programs for the application of specific methodological techniques (i.e., video tutorials, toolkit, dedicated lessons with experts) [11, 24]; (iv) the validation of the collected data by researchers/specialists before being transferred to national and international open access biodiversity repositories [25]. These points are of crucial importance to reach a wider audience and to ensure the reliability of the collected data [26].

Despite the strengths of MCS initiatives have been recently reviewed and highlighted in the literature [13, 14], common challenges are limiting the promotion of MCS projects and their long-term commitment in the diving community, even compromising the scientific credibility of these projects. Additionally, the lack of continuity and experts in the training process, as well as the use of non-standardized protocols, generally lead to a loss of attractiveness for divers in such activities through time. This lack of continuity is the main obstacle towards exploiting the leverage of MCS in the diving system, and integrating MCS activities and data to support effective management and conservation plans. In this context, we propose a more structured MCS approach based on three pillars, which create a circular win-win-win model enhancing the optimization and the full development of MCS. By involving divers in the scientific endeavors, our approach might lead to holistic and community-driven solutions for environmental challenges. The three pillars align with the principles of open innovation, sustainability, and participatory governance, being key aspects of the Quintuple Helix Model [27].

## Main text

### Pillar I: MCS as a tool for the site valorization

Scientific research is usually challenged by funding resources, drastically limiting the potential of extensive monitoring on large spatial and temporal scales [28]. The possibility to rely on a trained “team” results in a cost-effective and constant data collection [13], allowing to depict the consequences of abrupt or short-term events (e.g., storms, massive mortalities, species blooms) [29, 30], as well as the early detection of non-indigenous species (NIS) [21, 31].

The direct involvement of diving centers in the long-term monitoring of the health status of marine ecosystems through MCS initiatives allows for the engagement of a high number of divers [10]. These initiatives are, in most cases, promoted by Marine Protected Areas (MPAs) managers that, collaborating with the local stakeholders (i.e., diving centers), collect data to monitor the status of the species and habitats included within the MPA borders [32]. Generally, MCS actions target species of commercial interests, species included in European Directives (e.g., Habitat Directive, 92/43/EEC; Barcelona Convention) or reported in the International Union for the Conservation of Nature Red List (https://www.iucnredlist.org/), as well as rare and alien species [11, 13, 21, 22, 33–35]. Due to the unfolding of climate change and the increase of seawater temperatures, several NIS have colonized new habitats and, in some cases, replaced native species, significantly affecting the trophic webs [36, 37]. Therefore, the implementation of MCS projects focusing on all the above-mentioned aspects and also on the detection of the presence of direct and indirect anthropogenic impacts (e.g., mass mortality events, lost fishing gears) is of crucial importance to promptly adapt management strategies when necessary [38].

**Fig. 1:**
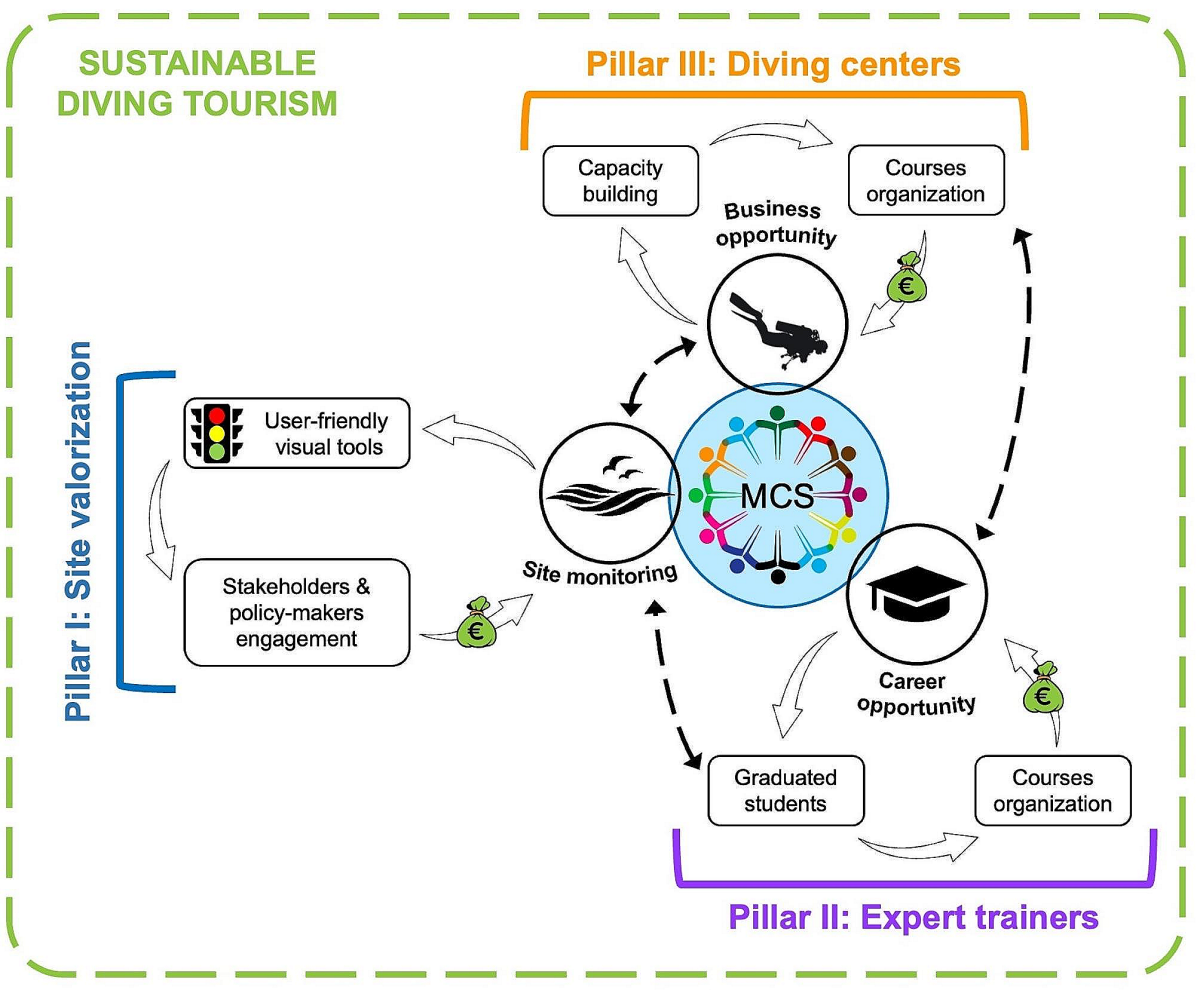
Schematic view of the three Marine Citizen Science (MCS) pillars bringing to sustainable diving tourism: the importance of the site valorization through monitoring performed with MCS activities (Pillar I), the career opportunity for expert trainers (e.g., marine biologists) with their involvement in such activities (Pillar II), and the business opportunity represented by these initiatives for diving centers (Pillar III)

If the same approach is applied outside MPAs, it will provide: (i) at local scale, precious data for the authorities to support evidence-based management, and (ii) at regional/national levels, the framework for policymakers to design potential new MPAs under different schemes, thus representing a powerful tool for the implementation of tailored conservation measures [26]. Overall, MCS initiatives can lead to enchance both the site valorization from a naturalistic point of view, and the preservation of the cultural heritage, boosting the attractiveness of the area for tourism (Fig. 1).

### Examples of the use of MCS for marine habitat conservation and management

The implementation of a user-friendly visual tools to present the results of the volunteers’ monitoring is paramount to communicate and inform policymakers and managers (not necessarily having specific expertise in the field) on the health and the ecological status of coastal marine ecosystems.

#### Example 1

The Interreg Med MPA Engage project (“Engaging Mediterranean key actors in Ecosystem Approach to manage Marine Protected Areas to face Climate change”) was funded to support Mediterranean MPAs as nature-based solutions to face climate change. The project gathered marine scientists, conservationists and MPAs managers working together to develop adaptation and mitigation plans against climate change. In this framework, 11 standardized protocols were developed, three specifically designed for citizen scientists after dedicated trainings. These protocols target the presence of mass mortalities of emblematic species (e.g., gorgonians, sponges, bryozoans), the presence of fish indicators of the Mediterranean tropicalization and meridionalization [39, 40], and the health status of the bivalve *Pinna nobilis* [24]. Related to them, a specific Data Reporting tool was developed in the PowerBi software and distributed to the MPA managers involved in this project. This application provides, using a traffic light visualization, the results of the monitoring, with red and green corresponding to a high or low impact, respectively (Fig. 1).

#### Example 2

Reef Check (RC) Italia (www.reefcheckmed.org) is a scientific non-profit organization established in 2008, dedicated to the protection and monitoring of Mediterranean coastal habitats. RC Italia developed the Mediterranean Underwater Coastal Environmental Monitoring protocol (RCMed U-CEM), targeting 13 habitats and 43 taxa, including protected and easy-to-identify species, species sensitive to impacts and climate change, and NIS. The MedSense index is a biotic index based on open data collected under the RCMed U-CEM, and available as a free user-friendly QGIS plugin. It converts data collected by trained volunteers focusing on 25 selected species into an effective monitoring tool for the Mediterranean subtidal rocky coastal habitats; similarly to the previous example, it provides a traffic light visualization of the environmental status of these habitats (Fig. 1). The index was specifically developed to increase the awareness of stakeholders and managers, to identify the main pressures acting in the targeted habitats, and to support them in the implementation of appropriate conservation measures and in communicating the results of their actions [33].

### Pillar II: MCS as a new career opportunity for graduated students

SCUBA divers involved in MCS initiatives may come from very diverse educational backgrounds, usually presenting no qualification in marine-related subjects [1, 13]. Nevertheless, their high interest in the marine environment makes them more prone to receive dedicated trainings, ensuring the collection of trustworthy data [17], only gathered if an expert trainer is involved in this phase.

Newly graduated students in marine sciences (e.g., marine biology and ecology, environmental and natural sciences) might be interested in doing research, but not necessarily in an academic framework, or they might be still uncertain about their future [41]. This might induce them to find employment in diving centers, to couple their passion for SCUBA diving and research, or to temporarily be employed to improve their diving skills while finding their path. Therefore, they may represent the ideal candidates to become trainers of MCS initiatives, representing a further career opportunity for the recent graduate (Fig. 1). However, most of the time, graduated students end up being employed only as diving staff since most diving centers still do not foresee the potential of these activities to boost their business (see Pillar III) [42].

### Examples of the use of MCS for career development

The proposal of MCS courses by expert and skilled trainers usually leads to successful initiatives involving a high number of trained volunteers willing to contribute to the monitoring and protection of the underwater environment (see Pillar I) [13].

#### Example 1

**Fig. 2:**
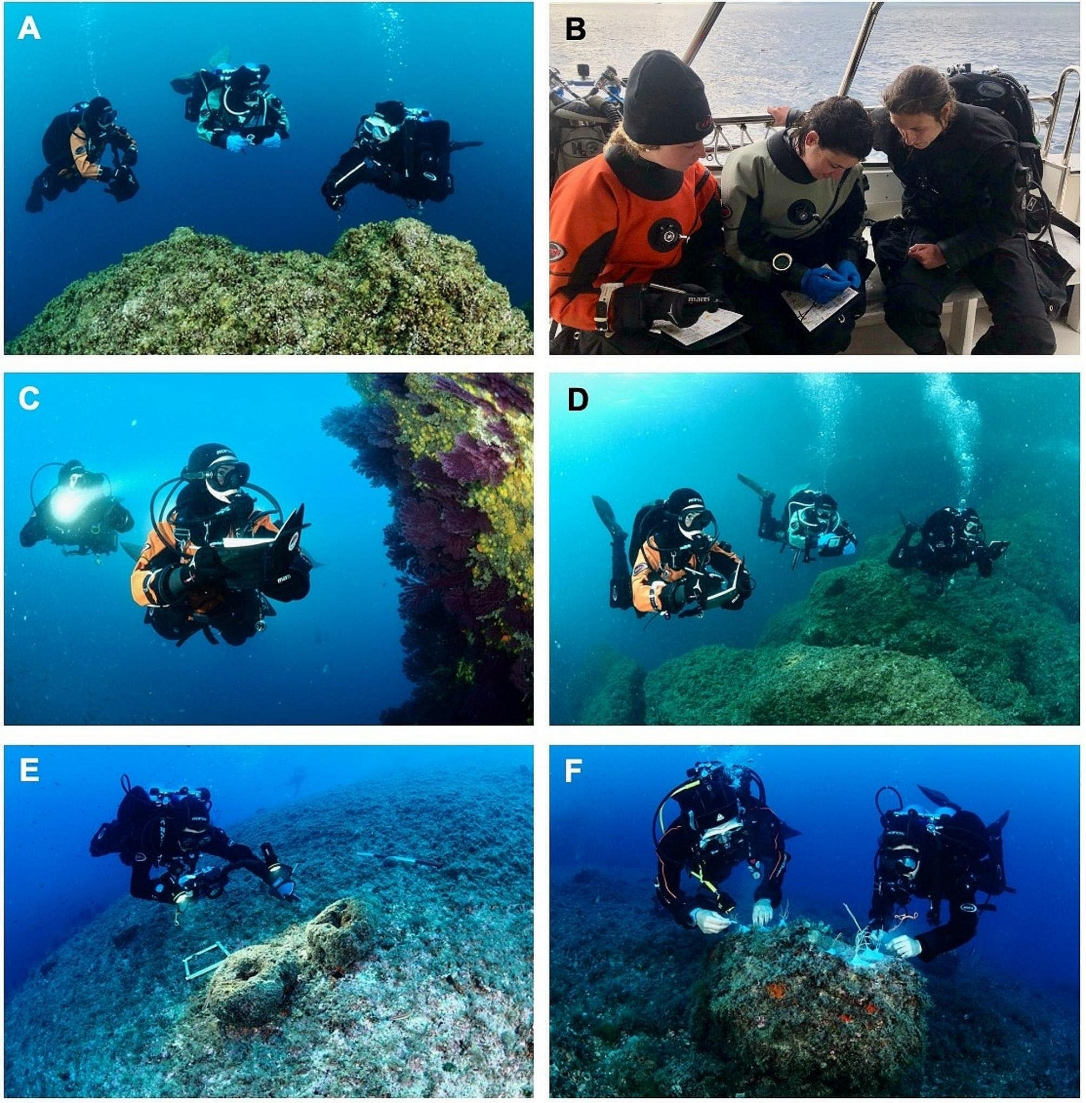
**(A-B)** A Reef Check Italia trainer supporting volunteers in the data collection and species identification. Volunteers involved in the MCS activities applying the MPA Engage **(C)** “Assessment and monitoring of mass mortality”, **(D)** “Fish visual census of climate change indicators”, **(E)** “Structure from Motion-Photogrammetry as a monitoring tool for benthic habitats structure and dynamics” protocols, and **(F)** transplanting corals of opportunity for restoration purposes

To be involved in the implementation of the RC Italia RCMed U-CEM protocol, volunteers must possess the Eco-diver certification delivered after their participation in a course held by a RC trainer [11, 33]. To become a RC trainer, an already certified Eco-diver must: (i) participate to a dedicated preparation to acquire the needed expertise to teach the protocol, (ii) take an exam and (iii) organize a first course independently. Courses usually last one/two days, including theoretical and practical activities, ending with a final exam and the upload of the data. The theoretical part is focused on the presentation of the RC organization, on the application of the RCMed U-CEM protocol and on how to gather the required data; while the practical activity is dedicated to the data collection with the trainer assisting volunteers, solving potential doubts and uncertainties (e.g., species identification) (Fig. 2A-B).

#### Example 2

Observadores del Mar (https://www.observadoresdelmar.es/) was created in 2012, and, up to date, more than 50 researchers from various Mediterranean research facilities are involved in the coordination of the objectives of the foundation, related to the conservation of Mediterranean marine ecosystems affected by anthropogenic pressures [25]. With the involvement of diving centers and non-governmental organizations, Observadores del Mar promotes courses, led by marine scientists, which include two days of training and supervised goal-oriented dives followed by the upload of the data on a dedicated platform; data are then validated by the researchers of the organization. At the end of the course, all volunteers are asked to take a qualitative survey to assess their overall satisfaction, as well as their perception about the marine environment after the dedicated training [25]. Additionally, Observadores del Mar is involved in the training of diving guides carrying out their activities inside Catalan MPAs.

### Pillar III: MCS as a business opportunity for diving centers

The consequences of the current climate emergency affecting marine communities are becoming more and more evident, especially for coastal communities that, living in close contact with the sea, are the first witnesses of these changes [24]. In this sense, diving centers can greatly contribute to the early detection of these effects once they have the proper awareness, knowledge, and tools. Therefore, they might be interested in directly participating in MCS initiatives and/or courses, acknowledging the importance of their capacity building [43].

Acquiring these skills might lead diving centers to organize their own courses, resulting in further economic incomes (Fig. 1) [43]. The presence in the staff of a marine scientist could facilitate this process (see Pillar II): offering courses led by experts in the field may reward volunteers with a high degree of gratification, especially when the application of specific techniques is involved [1], being able to apply these methods independently in the future. Additionally, trainers should receive remuneration for their work and effort, since it has been demonstrated that underpaid or exploited trainers do not deliver successful courses, ending with negative feedback and the loss of product value [16].

Transmitting knowledge on the marine environments together with increasing the awareness on the risks threatening their communities lead to a higher sensitivity of divers who become more conscious of the need for a responsible diving. In this sense, diving centers may play a crucial role in promoting a sustainable diving tourism. The simple deseasonalization of the diving activities performed during the entire year may: (i) result in a continuous monitoring of the sites usually frequented by divers, potentially recording anthropogenic and natural impacts in the long-term (see Pillar I); (ii) represent a constant source of income for the diving centers throughout the year [16, 44, 45]. Furthermore, the desire to be involved in MCS programs may encourage to improve personal diving skills, leading beginner divers to follow new diving courses.

### Examples of the use of MCS as a potential economic income for diving centers

MCS initiatives may be promoted by diving centers, particularly concerned about the changes that our seas are currently facing, only after having participated actively in specific training to acquire the needed knowledge on the topic.

#### Example 1

In the framework of the MPA Engage project, a specific training program, the “Basic Research Operator” (BRO), was co-designed by the Polytechnic University of Marche (Ancona, Italy), the Professional Association of Diving Instructors (PADI) and the European Divers Alert Network (DAN EU) to provide new skills to diving centers. Thanks to its structure, the course aimed to represent a new business opportunity for diving operators as well as a chance for recreational divers to contribute to MCS initiatives. The program included an initial training for diving centers owners with the provision of a manual explaining in detail various MCS protocols to monitor the conservation status of the main marine coastal habitats. Video tutorials were also provided to support the subsequent training of volunteers, including the procedures to follow for the data upload on dedicated repositories, to share the collected ecological data. All the training material was available on the DAN e-learning platform to guide the diving centers owners’ preparation. During the spring-summer 2020 and 2021, 18 events were organized in different countries (Italy, Croatia, Malta, and Spain) creating a network of training centers for passionate divers willing to contribute to the monitoring of Mediterranean habitats, with the BRO being presented to a total of 298 divers (including diving instructors, recreational and technical divers). After two years, this initiative was still active only where marine scientists were actively involved, confirming their key role to guarantee the long-term interest towards MCS projects (e.g., Asinara Camp, https://www.i7mari.it/asinara-camp-national/), while a general decrease in the level of participation was documented in all the other cases.

#### Example 2

Reef Alert Network (RAN; https://www.reefalert.org/) is a non-profit organization founded by a group of passionate underwater explorers, marine scientists and diving center owners, with the aim of shaping a new generation of divers capable of supporting science in the monitoring and recovery of the most threatened habitats in the Mediterranean Sea. To this purpose, the “Conservation Diver” course (https://portofinodivers.com/en/marine-conservation-volunteering.html) was created in 2022 in collaboration with the Polytechnic University of Marche. The course, held by marine biologists, focuses on the implementation of four monitoring protocols and one restoration action, particularly suitable to be applied by volunteers. The monitoring protocols include the RCMed U-CEM, and three MPA Engage protocols, specifically “Assessment and monitoring of mass mortality”, “Fish visual census of climate change indicators” and “Structure from Motion-Photogrammetry as a monitoring tool for benthic habitats structure and dynamics” (Fig. 2C-E) [24]. The restoration activity focuses on the use of transplantation techniques to save the so-called “corals of opportunity” (i.e., corals detached due to natural or anthropogenic impacts) from displacement and/or tissue damage, reattaching them with the use of a two-component epoxy (Fig. 2F) (see [46] for the method). The course lasts for five days of intensive theoretical training followed by goal-oriented dives applying the above-mentioned protocols.

Since 2022, four Conservation Diver courses were held, with 27 volunteers trained. The trained divers contributed with over 40 RCMed U-CEM surveys, 40 visual census of the mass mortality assessment protocol, resulting in the health assessment of more than 4,000 gorgonian colonies, and 80 fish visual census. The photogrammetry protocol is currently helping to monitor the habitat complexity of a *facies* of the precious red coral, *Corallium rubrum*, and of the keratose sponge, *Sarcotragus foetidus*, both highly threatened by the current climate anomalies. This collaboration resulted in the publication of scientific papers [47, 48], highlighting once more the importance of engaging passionate divers in the scientific research. In addition, the restoration efforts were focused on gorgonian species, especially on four species, *Leptogorgia sarmentosa*, *Eunicella singularis*, *E. cavolini* and *Paramuricea clavata*, leading to the transplantation of more than 40 fragments/colonies.

## Conclusions

It is widely accepted that MCS is a powerful tool either for data gathering and for the education of new generations of divers to a more responsible diving, helping to safeguard endangered marine ecosystems [10, 16]. This is reflected also on the rise of the number of publications on the topic. In fact, searching on the Scopus database using the query “marine citizen science”, a total of 899 documents are obtained, of which only 131 published before 2017, thus resulting in an increase of six times in the number of publications in the last 5 years (768 documents). This trend points out a general growth in the scientific interest on MCS, with the use of standardized monitoring protocols, the quality control, and the engagement of the public [25], opening new job opportunities for marine scientists with a strong attraction toward the diving sector. The opportunity to collect reliable data by trained volunteers is paving the way to a new approach for the diving market, but it is also fundamental to guarantee high scientific standards.

The three pillars here presented show a different perspective on the application of MCS, which may trigger a sort of “autocatalytic” system, that benefits the stakeholders involved (i.e., diving centers and trainers), with a source of income and business opportunity, and the policymakers, who obtain data for site protection, valorization and management [26, 38, 42]. When the pillars are well connected, they represent a win-win-win solution for all the users, leading to the creation of a more sustainable diving tourism, which protect the natural environment and its cultural value through long-term monitoring.

The importance of CS is well acknowledged even at European level, with the compilation of the best practices highlighting the crucial role of CS initiatives in complementing the environmental reporting, always preceded by a proper training to ensure a high quality data collection [49]. According to these guidelines, the three pillars here described fits well into the Blue Economy strategy and the Ocean Literacy approach, promoting the Sustainable Development Goal 14 (*Life Below Water*) of the Agenda 2030, which mandates for the conservation and sustainable use of marine resources and includes all the “actors” in play (i.e., touristic stakeholders, policy makers) [50–52]. In this sense, the promotion of MCS activities can sustain the tourist sector, particularly the eco-tourism, enhancing conservation and supporting local communities [12]. The recent approval of the Restoration Law is another tessera in the complex mosaic of habitat protection and conservation that can receive important contribution also from MCS initiatives.

Even though this new approach reported Mediterranean case studies, the same path can be followed in every coastal area of the world, given that the solid bases to build the three pillars exist. This will guarantee a potential long-term application of a community-based approach in alignment with the Quintuple Helix Model, demanding a socioecological transition of society and economy through eco-innovation and sustainable development [27].

## Data Availability

No datasets were generated or analysed during the current study.

## Abbreviations

BRO: Basic Research Operator
CS: Citizen Science
DAN EU: European Divers Alert Network
MCS: Marine Citizen Science
MPA: Marine Protected Area
NIS: Non-indigenous species
PADI: Professional Association of Diving Instructors
RAN: Reef Alert Network
RC: Reef Check
RCMed U-CEM: RC Italia developed the Mediterranean Underwater Coastal Environmental Monitoring
